# Sexual orientation and psychotic-like experiences among Chinese college students: the role of gender

**DOI:** 10.3389/fpsyt.2023.1139484

**Published:** 2023-09-08

**Authors:** Dali Lu, Zaihua Qing, Ying Tu, Xiaoqun Liu

**Affiliations:** ^1^Department of Pediatric Psychology, Shenzhen Longhua Maternity and Child Healthcare Hospital, Shenzhen, China; ^2^Hunan University of Finance and Economics, Changsha, Hunan, China; ^3^Department of Maternal and Child Health, Xiangya School of Public Health, Central South University, Changsha, Hunan Province, China

**Keywords:** sexual orientation, psychotic-like experiences, college students, hallucinatory experiences, delusional experiences

## Abstract

**Objective:**

The mental health of sexual minorities has received increasing attention, but there are few studies on the risk of psychotic-like experiences (PLEs) among sexual minorities. The purpose of this study is to explore the relationship between different sexual orientations and PLEs among college students and the moderating effect of gender.

**Methods:**

A total of 4,460 college students from seven provinces participated in this cross-sectional survey. The χ^2^ test and logistic regression were used to investigate the relationship between sexual orientation and PLEs.

**Results:**

Of the participants, 4.9% identified as bisexual, 1.1% as lesbian/gay, and 5.6% were questioning/unsure; 60.1% of the sample experienced at least one PLE item, 59.2% reported delusional experiences (DEs), and 20.6% had hallucinatory experiences (HEs). Compared with heterosexual college students, bisexual and questioning students showed a higher risk of PLEs, DEs, and HEs, and lesbian/gay students showed a higher risk of HEs. Stratified analysis indicated that sexual orientation was significantly associated with PLEs only for female college students.

**Conclusion:**

Sexual orientation is a predictive factor of PLEs. In particular, different sexual minority subgroups show the different effects on PLEs between male and female college students. Mental health interventions for PLEs could employ distinct strategies based on different sexual orientations and gender disparity.

## 1. Introduction

Sexual minorities are individuals who identify as non-heterosexuals based on their self-identification (such as lesbian, gay, bisexual, and questioning\unsure), sexual behavior, and/or sexual attraction (1). Many studies have found that sexual minorities are more likely to experience negative mental health outcomes than heterosexuals, e.g., problem behaviors (alcohol, tobacco use etc.) (2, 3), especially emotional problems (depression, anxiety, etc.) (4) and even suicidal risk (no suicide self-injury, suicide behavior, etc.) (5). According to Meyer’s minority stress model, the reason why sexual minorities suffer more negative psychological outcomes is that they suffer more discrimination, harassment, victimization, expectations of rejection, identity concealment, and internalized heterosexism (6). Therefore, the mental health of sexual minorities deserves more attention. However, existing studies mainly focus on Western countries (7, 8). Chinese people are deeply influenced by collectivist culture (9) and have a relatively low acceptance level of individualistic sexual minorities (10), and it was not until 2001 that homosexuality was formally removed from mental disorders (11). At the same time, Chinese traditional culture overemphasizes the continuity of the family bloodline and filial piety; it is regarded unfilial not to marry and have children (12). Therefore, the sexual minority youth are highly stigmatized and experience social discrimination from multiple people in their lives in this environment. A study showed that nearly a quarter of SMS (men who have sex with men) report that they have experienced stigma and discrimination, and 75% have concealed their identity by pretending they were not SMS (13). In recent years, some studies indicated that Chinese sexual minority individuals are at a greater risk of physical and mental health problems, but they mainly focused on gay men and adults, and there are few studies on college students (14, 15). However, college is considered a “sensitive period” for the development or exacerbation of mental health concerns (16), and increased bullying, discrimination, stigma, harassment, and other similar health disparities have been linked to increased mental health problems among sexual minority college students (17); therefore, it is necessary to further study the mental health of sexual minorities in the context of China.

Psychotic-like experiences (PLEs) are defined as experiences that resemble the positive symptom (such as delusional ideation or hallucinatory experiences) of psychosis, as encountered in clinical samples but which do not cause the levels of distress or impairment that lead to clinically significant distress, disability, or loss of functioning (18). Therefore, PLEs are also often called subclinical psychotic symptoms, which are common in non-clinical samples and mostly transient in nature (19). However, PLEs usually present as an important precursor for psychiatric disorders (20). Early positive symptoms with delusions and hallucinations interacting with environmental exposure could easily lead to the persistence of PLEs and eventually result in a higher risk of subclinical and clinical psychosis (21). Additionally, these PLEs are associated with an elevated risk of subsequent emotional and behavioral problems (22), and even suicidal ideation, suicide attempts, and suicide deaths (23). Multiple factors such as drug use (19), parental communication patterns (24), separation from parents (25), and personal factors (e.g., coping style and personality traits) (26), especially trauma (e.g., bullying, child abuse, and neglect) (27, 28), have been associated with PLEs. As noted, it is well known that sexual minorities often experience more trauma (e.g., exclusion, discrimination, and bullying) (29) because of their non-traditional sexual orientation, but whether they will experience more PLEs has not well been well studied. To date, only a few studies about the association between sexual orientation and psychosis are available. According to Gevondenet et al.’s study in the Netherlands (30), 39.1% of lesbian, gay, and bisexual (LGB) people have reported PLEs, compared with 16.5% of heterosexuals (OR 3.25, 95% CI 2.22–4.76), suggesting that sexual minority status is associated with a higher prevalence of psychotic symptoms in general population studies. Another study in England also showed that non-heterosexual orientation is positively associated with psychotic experiences (31). However, Oh considered that non-heterosexuality is differentially associated with certain PLEs (visual hallucinations or delusions), depending on the racial population being examined (32). Qi’s study also showed sexual minority status does not significantly predict probable psychosis but indicated a specific association between sexual minority status and paranoia when contrasted with auditory verbal hallucinations (33). Thus, more studies are needed in other contexts to better understand the association between sexual orientation and psychosis, particularly subclinical psychosis (PLEs), to reduce the risk of distressing psychotic experiences within sexual minority populations.

Different sexual minority groups may experience different pressures and mental health problems. For example, bisexuals are at the greatest risk of suicide in Mattia’s systematic review (34). Treating sexual minorities as a uniform population or overlooking a specific group (e.g., those who are questioning or exploring their sexual orientation) could mask the mental health risks faced by some sexual minority groups (35, 36); therefore, it is necessary to explore the disparity in PLEs between different sexual minority groups (lesbian/gay, bisexual, and questioning). Furthermore, there may be gender differences in the association between sexual orientation and PLEs. The existing literature has shown that female sexual minorities predict more depressive symptoms than sexual minority men (8, 37). Another study has also shown that gay men and bisexual women exhibit a 2-fold greater occurrence of schizophrenia and psychotic illness or episodes than their heterosexual equivalents (38). However, Jacob’s study revealed that gender was not a significant effect modifier in the sexual orientation-psychotic experiences relationship (31). Therefore, the effect of different forms of sexual orientation on PLEs between males and females warrants further research. This study has two objectives: (1) to investigate the prevalence of PLEs among different types of sexual minorities in Chinese college students, and (2), to examine the effect of the interaction between sexual orientation and gender on PLEs.

## 2. Methods

### 2.1. Procedures and participants

A multistage convenient sampling was adopted in this cross-sectional and multicenter survey from April to June, 2022. First, seven provinces (Hunan, Jiangxi, Hubei, Jilin, Guangdong, Guizhou, and Shandong) were selected from regions of different economic levels in China, and then three or four universities of different types were invited to participate in the survey in each province. Finally, 180–260 students from each sample university were invited to participate in the survey. A total of 24 universities, including three excellent undergraduate colleges (4 years and above), 11 general undergraduate colleges, and six vocational colleges (3 years), agreed to participate in the survey. These schools represent a range of university environments in China.

The inclusion criteria for participation in this study were as follows: full-time college students, ability to complete the web-based questionnaire on a smartphone by themselves, and voluntary participation. The exclusion criteria were self-reported history of diagnosed schizophrenia disorder or did not complete the questionnaire independently. A total of 4,736 college students agreed to participate in the survey, 246 failed to submit the complete questionnaire, 30 were excluded because of their history of a psychiatric condition, and 4,460 were finally included in the analysis.

This study has been approved by the Xiangya Public Health Ethics Committee of Central South University. Before the survey, the participants were informed about the main content of the survey and its anonymity. Then, the students decided whether to participate or not. If they agreed to participate, they completed the questionnaire online in approximately 20 min. Students could consult our investigators at any time if they were not clear about the questions. At the end of the questionnaire, we showed the participants a page about ways of seeking help if they had serious mental health problems.

### 2.2. Instruments

#### 2.2.1. Sexual orientation

Based on previous studies, this study used the item, “Which of the following best describes you?” to examine participants’ sexual orientation. The responses included: (1) heterosexual; (2) Lesbian/gay; (3) bisexual; and (4) questioning or unsure. The students who choose (2), (3), or (4) were defined as sexual minorities (39, 40).

#### 2.2.2. Community assessment of psychic experiences

Konings et al. created the Community Assessment of Psychic Experiences (CAPE) (41), which includes 42 items to measure positive (20 items), negative (14 items), and depressive (eight items) psychotic symptoms on a frequency scale (1 = never, 2 = sometimes, 3 = often, and 4 = nearly always) and a distress scale (1 = not distressed, 2 = a bit distressed, 3 = quite distressed, and 4 = very distressed). The Chinese mainland version of CAPE has been translated and validated for some pilot studies and demonstrated good reliability and validity (42, 43).

Psychotic-like experiences were measured using the positive subscale of CAPE. The subscale consists of 20 items measuring PLEs. All items were obtained from the scale derived from Peters et al.’s Delusions Inventory (PDI-21) (44). As some of the items were very long and common, we chose eight [six items were related to delusional experiences (DEs), and two were related to hallucinatory experiences (HEs)] of them to reflect PLEs based on previous research (45, 46). The short version of the eight-item CAPE has good reliability and validity in the Chinese population (18, 47), and can evaluate the PLEs well. The respondents who chose sometimes or above for any items were considered to experience any PLEs, and the participants who chose often or nearly always for any items were denoted as having frequent PLEs.

#### 2.2.3. Demographic and confounding variables

Demographic information and confounding variables in this study include gender (1 = male and 2 = female), grade (1 = 1st, 2 = 2nd, 3 = 3rd, and 4 = 4th), family location (1 = rural and 2 = city), race (1 = Han and 2 = minority), mother’s education level (1 = primary or junior high school, 2 = high school, and 3 = college or above), self-reported family financial situation (1 = poor, 2 = medium, and 3 = rich), relationship with classmates (1 = poor, 2 = average, and 3 = good), relationship with teachers (1 = poor, 2 = average, and 3 = good), and relationship with parents (1 = poor, 2 = average, and 3 = good).

### 2.3. Data analysis

All data were inputted by EpiData 3.1 and analyzed by spss 20.0. First, descriptive statistics were used to present the general distribution of all variables, including the demographic characteristic, confounding variables, the distributions of different sexual orientation, and the prevalence of PLEs. Then, the χ^2^ test and logistic regressions were performed to examine the associations between different sexual orientation and PLEs. To examine gender disparity, interaction analysis was conducted using the χ^2^ test and hierarchical analysis was performed using logistic regressions (40, 48). In the adjusted logistic regression analysis, we controlled for all demographic and confounding variables (e.g., grade, race, family location, mother’s education, financial situation, relationship with parents, relationship with teachers, and relationship with classmates) that were significant in the χ^2^ test. The association was reported via odd ratios (OR) and 95% confidence intervals (95% CI). *p* values less than 0.05 were considered statistically significant.

## 3. Results

### 3.1. Sample characteristics and the prevalence of sexual minorities and PLEs among the participants

The mean age of the 4,460 participants was 19.48 ± 1.6, ranging from 18 to 24 years old. There were 3,073 girls (68.9%) and 1,387 boys (31.3%) and 92.3% of the participants were Han Chinese. Regarding sexual orientation, 88.4% of the participants reported being heterosexual, 5.6% were questioning, 4.9% were bisexual, and 1.1% were lesbian/gay. In terms of PLEs, 60.1% of the sample experienced at least one PLE item (59.2% for DEs and 20.6% for HEs) and only 14.3% experienced frequent PLEs (13.9% for frequent DEs and 3.0% for frequent HEs). The sample characteristics and the prevalence of sexual minorities are shown in Table 1.

**Table 1:** Sample characteristics and sexual orientation among the participants (*n*[%]).

Variables	Total (*n* = 4,460)
Gender	
Male	1,387 (31.1)
Female	3,073 (68.9)
Grade	
1st	2,285 (51.2)
2nd	1,245 (27.9)
3rd	688 (15.4)
4th	242 (5.4)
Race	
Han	4,116 (92.3)
Minority	344 (7.7)
Family location	
Rural	2,726 (61.1)
City	1,734 (38.9)
Mother’s education	
Primary school and below	2,669 (59.8)
Junior and senior school	1,091 (24.5)
College and above	625 (14)
Motherless	75 (1.7)
Family financial situation	
Low	655 (14.7)
Middle	3,402 (76.3)
High	403 (9.0)
Relationship with classmates	
Good	2,597 (58.2)
Average	1,833 (41.1)
Poor	30 (0.7)
Relationship with teachers	
Good	1,691 (37.9)
Average	2,733 (61.3)
Poor	36 (0.8)
Relationship with parents	
Good	3,523 (79)
Average	913 (20.5)
Poor	24 (0.5)
Sexual orientation	
Heterosexual	3,941 (88.4)
Questioning	250 (5.6)
Bisexual	220 (4.9)
Lesbian/gay	49 (1.1)

### 3.2. Comparison of psychotic-like experiences in demographics and confounding variables

The students in lower grades and from rural areas scored higher on the total rate of PLEs and the other two dimensions (DEs and HEs) than the students in higher grades and from the city (*p* < 0.001). The participants who perceived that their families were wealthy and had good relationships with classmates, teachers, and parents reported fewer PLEs, DEs, and HEs (*p* < 0.001). In addition, students from minority ethnic groups experienced more HEs (*p* < 0.05), and participants whose mothers had a higher education reported fewer PLEs and DEs (*p* < 0.01). The results are presented in Table 2.

**Table 2:** Comparison of psychotic-like experiences in demographics and confounding variables.

Variables	DEs	HEs	PLEs
Grade			
1st	1,406(61.5)	519(22.7)	1,429(62.5)
2nd	736(59.1)	253(20.3)	750(60.2)
3rd	362(52.6)	115(16.7)	367(56.2)
4th	135(55.8)	32(13.8)	136(56.2)
χ^2^	18.66^***^	20.69^***^	20.32^***^
Race			
Han	2,422(58.8)	833(20.2)	2,463(59.8)
Not Han	217(63.1)	86(25.0)	219(63.7)
χ^2^	2.36	4.4^*^	1.94
Family location			
Rural	1,656(60.7)	598(21.9)	1,684(61.8)
City	983(56.7)	321(18.5)	998(57.6)
χ^2^	7.23^**^	7.60^**^	7.88^**^
Mother’s education			
Primary school and below	1,629(61.0)	565(21.2)	1,655(62.0)
Junior and senior school	634(58.1)	220(20.2)	645(59.1)
College and above	331(53.0)	123(19.7)	337(53.9)
χ^2^	14.31^**^	0.94	14.45^**^
Family financial situation			
Low	432(66.0)	190(29.0)	440(67.2)
Middle	2,007(59.0)	661(19.4)	2,037(59.9)
High	200(49.6)	68(16.9)	205(50.9)
χ^2^	27.71^***^	34.57^***^	28.07^***^
Relationship with classmates			
Good	1,412(54.4)	447(17.2)	1,438(55.4)
Average	1,204(65.7)	451(24.6)	1,221(66.6)
Poor	23(76.7)	21(70.0)	23(76.7)
χ^2^	60.77^***^	8.94^***^	60.08^***^
Relationship with teachers			
Good	849(50.2)	285(16.9)	865(51.2)
Average	1,764(64.5)	615(22.5)	1,789(65.5)
Poor	26(72.2)	19(52.8)	28(77.8)
χ^2^	9.45^***^	43.34^***^	93.90^***^
Relationship with parents			
Good	2,009(57.0)	660(18.7)	2,043(58.0)
Average	608(66.6)	244(26.7)	617(67.6)
Poor	22(91.7)	15(62.5)	22(91.7)
χ^2^	38.03^***^	54.19^***^	37.82^***^

DEs, delusional experiences; HEs, hallucinatory experiences; PLEs, psychotic-like experiences. ^*^p < 0.05, ^**^p < 0.01, and ^***^p < 0.001.

### 3.3. Prevalence of PLEs by sexual orientation and gender

As seen in Table 3, the incidences of PLEs (χ^2^ = 24.59), DEs (χ^2^ = 21.99), and HEs (χ^2^ = 47.81) were significantly different among college students with different sexual orientations. The prevalence of PLEs was higher among the questioning and bisexuals, followed by lesbian/gay people and heterosexuals. In addition, gender differences were significant in the prevalence of PLEs (χ^2^ = 37.81), DEs (χ^2^ = 38.02), and HEs (χ^2^ = 6.63). The DEs and PLEs among girls were higher than boys, while the HEs among girls were lower than boys. Moreover, there were significant gender differences in the relationship between sexual orientation and PLEs (χ^2^ = 58.32), DEs (χ^2^ = 56.55), and HEs (χ^2^ = 56.64). The specific differences in the prevalence of PLEs by sexual orientation×gender is shown in Table 3.

**Table 3:** Prevalence of PLEs by sexual orientation and gender.

	DEs	χ^2^	HEs	χ^2^	PLEs	χ^2^
	*N* (%)		*N* (%)		*N* (%)	
Sexual orientation		21.99^***^		47.81^***^		24.59^***^
Heterosexual	2,284 (58.0)		258 (19.2)		2,319 (58.8)	
Questioning	171 (68.4)		87 (34.8)		176 (70.4)	
Bisexual	154 (70.0)		55 (25.0)		156 (70.9)	
Lesbian/gay	30 (61.2)		19 (38.8)		31 (63.3)	
Gender		38.02^***^		6.63^**^		37.81^***^
Male	727 (52.4)		318 (22.9)		741 (53.4)	
Female	1,912 (62.2)		601 (19.6)		1,941 (63.2)	
Sexual orientation × gender		56.55^***^		56.64^***^		58.32^***^
Male × heterosexual	655 (51.5)		278 (21.8)		668 (52.5)	
Male × questioning	37 (63.8)		21 (36.2)		37 (63.8)	
Male × bisexual	22 (68.8)		10 (31.3)		22 (68.8)	
Male × lesbian/gay	13 (54.2)		9 (37.5)		14 (58.3)	
Female × heterosexual	1,629(61.1)		480 (18.0)		1,651 (61.9)	
Female × questioning	134 (69.8)		66 (34.4)		139 (72.4)	
Female × bisexual	132 (70.2)		45 (23.9)		134 (71.3)	
Female × lesbian/gay	17 (68.0)		10 (40.0)		17 (68.0)	

DEs, delusional experiences; HEs, hallucinatory experiences; PLEs, psychotic-like experiences. ^*^p < 0.05, ^**^p < 0.01, and ^***^p < 0.00.

### 3.4. Association between sexual orientation and PLEs in male and female college students

In the total sample, unadjusted regression analysis (Table 4) showed that the questioning and bisexual college students had a higher risk of PLEs, DEs, and HEs than their heterosexual peers, while lesbian/gay people only had a higher risk of HEs (OR = 2.66, 95%: 1.49–4.75). After adjusting for grade, race, family location, mother’s education, family economic status, relationships with classmates, teachers, and parents, questioning (AOR = 1.63, 95% CI: 1.22–2.19) and bisexual (AOR = 1.65, 95% CI: 1.21–2.24) participants reported a higher prevalence of PLEs and more DEs and HEs than heterosexual participants. In the female sample, the results of the unadjusted regression analysis were similar to that of the total sample, but the adjusted regression analyses showed that the higher risk of bisexual and lesbian/gay people experiencing HEs than heterosexual people was no longer significant. In the male sample, adjusted regression analysis showed there were no significant differences in the prevalence of PLEs, HEs, and DEs between different sexual orientation groups.

**Table 4:** Multivariate binary logistic regression of sexual orientation on psychotic-like experiences.

	Model 1			Model 2		
	DEs	HEs	PLEs	DEs	HEs	PLEs
	OR (95%C)	OR (95%C)	OR (95%C)	AOR (95%C)	AOR (95%C)	AOR (95%C)
Total
Heterosexual (reference)	1					
Questioning	1.57 (1.19–2.07)^**^	2.24 (1.71–2.94)^**^	1.66 (1.26–2.20)^***^	1.53 (1.15–2.04)^**^	2.03 (1.53–2.70)^**^	1.63 (1.22–2.19)^**^
Bisexual	1.69 (1.26–2.27)^***^	1.40 (1.02–1.92)^*^	1.71 (1.27–2.30)^***^	1.63 (1.21–2.21)^**^	1.38 (1.00–1.91)^*^	1.65 (1.21–2.24)^**^
Lesbian/gay	1.15 (0.64–2.04)	2.66 (1.49–4.75)^**^	1.21 (0.67–2.16)	0.91 (0.50–1.66)	1.88 (1.00–3.51)^*^	0.94 (0.51–1.73)
Male
Heterosexual (reference)	1					
Questioning	1.66 (0.96–2.87)	2.03 (1.17–3.53)^*^	1.60 (0.92–2.76)	1.67 (0.93–2.98)	1.67 (0.93–3.00)	1.6 (0.89–2.86)
Bisexual	2.08 (0.98–4.42)	1.63 (0.76–3.48)	1.99 (0.94–4.24)	2.06 (0.94–4.50)	1.86 (0.82–4.18)	1.99 (0.91–4.34)
Lesbian/gay	1.12 (0.50–2.51)	2.15 (0.93–4.96)	1.27 (0.56–2.88)	0.88 (0.37–2.08)	1.45 (0.56–3.73)	1.03 (0.43–2.44)
Female
Heterosexual (reference)	1					
Questioning	1.47 (1.07–2.03)^*^	2.39 (1.75–3.27)^***^	1.62 (1.17–2.24)^**^	1.40 (1.01–1.95)^*^	2.22 (1.61–3.08)^***^	1.55 (1.10–2.18)^*^
Bisexual	1.50 (1.09–2.88)^*^	1.43 (1.01–2.03)^*^	1.53 (1.10–2.12)^*^	1.44 (1.03–2.00)^*^	1.42 (0.99–2.02)	1.47 (1.05–2.05)^*^
Lesbian/gay	1.36 (0.58–3.15)	3.04 (1.36–6.81)^**^	1.31(0.56–3.04)	1.02 (0.43–2.42)	2.15 (0.92–5.03)	0.94 (0.40–2.24)

AOR, adjusted OR. ^*^p < 0.05, ^**^p < 0.01, and ^***^p < 0.001; DEs, delusional experiences; HEs, hallucinatory experiences; and PLEs, psychotic-like experiences.

Model 2 of DEs and PLEs: adjusted for grade, family location, mother’s education, financial situation, relationship with parents, relationship with teachers, and relationship with classmates.

Model 2 of HEs: adjusted for grade, race, family location, financial situation, relationship with parents, relationship with teachers, and relationship with classmates.

## 4. Discussion

The main purpose of this study was to investigate the relationship between sexual orientation and PLEs and compare PLEs among different types of sexual orientation groups (heterosexuality, lesbian/gay, bisexual, and questioning). To the best of our knowledge, only a few studies in England, the Netherlands, and the United States have examined the association between sexual orientation and psychosis. These few findings warrant further replication in other contexts, in which the conceptualizations and experiences of sexuality and psychosis can vary by population and culture. This is the first study to explore the relationship between sexual orientation and PLEs among the general Chinese college population. Additionally, this study expanded existing research by controlling a series of demographic variables and interpersonal variables (relationships with peers, teachers, and parents). Furthermore, this study aimed to explore the interaction between sexual orientation and gender with regard to the prediction of PLEs and compare gender differences in the association between sexual orientation and PLEs. It is expected that the results will provide more guidance for the mental health education of sexual minorities of different genders. Some interesting and meaningful results are obtained in this study.

First, in our study, the percentage of sexual minorities is 11.6%, which is similar to the recent study in China in 2022, but higher than the study in 2018, showing an increasing percentage of college students identifying themselves as a sexual minority over the years (11.6% in our study, 10.09% in 2022) (37), and 7.7% in 2018 (49). This trend suggests that sexual minority adolescents are becoming increasingly more open and comfortable to identify as a sexual minority instead of hiding their sexuality because of traditional morality, as in the past. The percentage of college students questioning their gender orientation in this study is 5.6%, which was higher than that in the United States study [3.9%; (50)]. The reasons for this disparity may be as follows: (1) different studies used different survey tools and criteria for defining people who question their sexual orientation; using a single item to determine sexual orientation may lead to heterogeneity in research results (51). (2) China’s long-standing conservative attitude toward sexual minorities may lead to a degree of self-concealment when gay, lesbian, and bisexual people report their sexual orientation.

Second, this study is the first to explore PLEs among sexual minorities in the context of Chinese culture; our findings replicated the results in other studies and added to the literature by showing the findings in non-Western people. In this study, all sexual minorities experienced a higher incidence of PLEs, DEs, and HEs than heterosexuals, although some of the differences were not statistically significant. For example, the incidence of HEs in the general population was 19.2%, whereas the incidence of HEs in the bisexual group was up to 38.8%. This is consistent with the findings of previous studies that showed higher rates of mental health problems (including PLEs) among sexual minorities (8, 33, 52). The experience of minority stress may be an important mechanism by which sexual minorities are at an increased risk of psychosis, i.e., sexual minorities will incur more social exclusion, homophobic pressure, and bullying, which can lead to trauma and psychiatric symptoms. Two studies have proved that childhood trauma, bullying, and experience of discrimination partly mediate the association between sexual minorities and psychotic symptoms (30) and non-affective psychotic disorder (53). In addition, the depersonalization and derealization associated with schizophrenia can be linked to doubts about one’s gender and sexual orientation (38); therefore, it is possible that PLEs precede beliefs of a non-heterosexual orientation. Another relevant factor for interpreting increased prevalence rates of PLEs within the sexual minority community may be substance use. Sexual minorities are more likely to experience substance use (54), which often leads to episodes of acute or chronic psychotic symptoms, including PLEs (55).

Third, this study expanded the existing studies by examining the PLE risk of different sexual minority groups (bisexual, gay/lesbian, and questioning). The results showed that bisexual college students were significantly more likely to experience PLEs, DEs, and HEs than heterosexual college students. This finding was similar to results obtained about other mental health concerns. For example, evidence suggests bisexual youths reported higher levels of depression, anxiety, and traumatic distress than heterosexual youths (56). The same studies found that bisexual youths are disproportionately impacted by peer victimization and subsequent suicidality compared with heterosexual youths (57, 58). Moreover, the systematic review found that bisexual individuals had a higher risk of suicide and exhibited more mental health problems than heterosexual, lesbian, and gay individuals. The reason that bisexual individuals have poorer mental health even when compared with gay and lesbian persons may be associated with minority stress (6) and internalized stigma (59) in general, and the unique experiences of “double discrimination” from both the gay/lesbian communities and heterosexual communities (60–63). In addition, bisexual people may experience more current adverse life events, greater childhood adversity, and a higher frequency of financial problems, as well as have less support from family, friends and the community (64, 65), which may contribute to adverse mental health outcomes in line with interpersonal-psychological theory (IPT). Furthermore, unlike former studies that typically focused on the comprehensive psychotic experience, we explored the relationship between sexual orientation and specific PLEs, including DEs and HEs. Questioning and bisexual people reported a higher prevalence of PLEs, DEs, and HEs than heterosexuals, and lesbian/gay people only reported a higher prevalence of HEs, which may suggest that there are different mechanisms between questioning and bisexual people and lesbian/gay people.

Consistent with past findings (66), we also found that questioning college students were at an increased risk of mental health problems. Specifically, they exhibited a significantly higher risk of PLEs, DEs, and HEs than heterosexual college students. The causes of mental health problems in questioning youths and their underlying factors remain unclear. In addition to societal stigmatization and high rates of bullying about their sexuality (66, 67), questioning youths may need more psychological energy to develop their sexual identity and explore their sexual attractions, which may limit the energy available for tackling other developmental challenges and place them at a higher risk of mental health challenges according to the bottleneck hypothesis (56, 68). Similar to bisexual youths, young questioning youths may experience stress due to limited social support and connection from the LGBQ (Lesbian, Gay, Bisexual, and Questioning) community due to their minority status within that community (67, 69). This finding underscores the importance of considering questioning youths as a distinct group.

In this study, lesbian/gay college students had a higher risk of HEs than heterosexual college students. This is consistent with the findings that lesbian/gay people experience more mental health problems, such as depression, anxiety, and suicide, than straight people, as well as psychotic symptoms (38, 70, 71). However, it is unclear why lesbian/gay college students did not appear to be comparatively more at risk of DEs and PLEs. The hypothesis that adversity can have specific effects on psychotic experiences may account for this to some extent (72). Previous studies have shown that sexual identity significantly predicts increased paranoia, whereas sexual identity does not significantly predict auditory verbal hallucinations (38). Additionally, a similarly study found that sexual minorities may be differentially associated with certain psychotic experiences [visual or auditory hallucinations and delusions (32)]. It is possible that the pathways by which sexual minority stress acts on delusions and hallucinations are different. In addition, there was a smaller percentage of lesbian/gay college students in our sample, which may also account for the null findings.

Finally, this study also found that the interaction between gender and sexual orientation had a significant effect on PLEs. Specifically, the risks of PLEs, DEs, and HEs for bisexual and questioning college students were significantly higher than those of heterosexual college students in female groups, whereas there was no significant association between PLEs, DEs, and HEs and sexual minority status in male groups. It is unclear why female sexual minorities have a higher risk of HEs, DEs, and PLEs than sexual minority males. It could be that female sexual minorities have less social support (73), are more susceptible to emotional problems (74), and experience more traditional sexual script pressures due to gender inequality (e.g., less sexual freedom, more restriction of sexual contacts, and shame for premarital sex among females) (40, 75–77). Studies have shown that female sexual minority youth (SMY) are more likely to be at risk of bullying, hopelessness, and suicide than their male counterparts (36, 78). A meta-analysis showed that female SMY were more likely to report depressive symptoms than male SMY (8). Additionally, a recent study in China found that lesbian and bisexual college students reported higher levels of depression than their male counterparts (37). All these similar studies support the findings of this study, but the small number of male college students in this study may yield some null findings. Additionally, the study in this field is very limited, not to mention other inconsistent findings that there were no significant gender differences in the relationship between sexual orientation and PLEs (31). Therefore, further studies are needed to ascertain the gender disparity and the potential factor that may account for it.

## 5. Strengths and limitations

The first strength of this study is the national sample, with a large sample size from seven provinces. Second, it is the first study to pay attention to PLEs among sexual minority Chinese college students. The major strength is the findings in the subgroups (heterosexual, lesbian/gay, bisexual, and questioning) and the analysis of the moderating effect of gender, which provided more comprehensive information about PLEs in different types of sexual minority college students and gender difference, which were not considered in previous studies. However, the findings from this study should be interpreted cautiously due to some limitations. First, this study used self-report questionnaires to obtain all the data, which may cause recall bias. Second, this study adopted cross-sectional design; therefore, the causal direction between variables cannot be concluded. Third, there was still a certain sample deviation, resulting in a small number of male participants. Therefore, the statistical effect may be reduced due to the small number of male students. Third, only a single item about sexual identity was used to measure sexual orientation; although this is common in current scientific practice (35), it still limited our evaluation of the PLEs of transgender, intersex, queer, pansexual, asexual, or polygamous individuals who belong to a broader sense of sexual minorities. Future studies need to take measures of sexual behavior or attraction into account to address the complexity of defining sexual minorities and obtain more accurate results. Fourth, although this study controlled some related variables and investigated the moderation of gender, the mediating factors (e.g., bullying, discrimination, child trauma, and shame) and other moderating factors (e.g., social support, resilience, and coping style) need to be further studied to explore the pathway or mechanism between sexual orientation and psychotic-like experiences. Finally, psychotherapeutic support or counseling may play a role in moderating or masking the symptoms under study; future studies should consider controlling for these moderators.

## 6. Conclusion

The findings of this study showed that the risk of PLEs in sexual minorities, especially in bisexual and questioning college students, was significantly higher than that in heterosexual college students. The relationship between lesbian/gay people and PLEs was related to the specific symptoms of PLEs, and the risk of PLEs in female sexual minorities was higher than that in male sexual minorities. Therefore, in future research and clinical practice, we should consider sexual minorities as different groups instead of aggregating the outcomes as a whole or ignore some certain groups, especially bisexual or questioning. Additionally, we should pay more attention to mental health problems, including PLEs, in sexual minority college students, especially in female sexual minorities.

## Data availability statement

The raw data supporting the conclusions of this article will be made available by the authors, without undue reservation.

## Ethics statement

The studies involving humans were approved by the Xiangya Public Health Ethics Committee of Central South University. The studies were conducted in accordance with the local legislation and institutional requirements. The participants provided their written informed consent to participate in this study.

## Author contributions

DL contributed to the study concept, statistical analysis, manuscript draft, and revision. ZQ and YT contributed to the data collection and statistical analysis. XL contributed to the study design and manuscript revision. All authors contributed to the article and approved the submitted version.

## Funding

This work was supported by the Social Science Fund of Hunan province “grant number is 22YBA013”.

## Conflict of interest

The authors declare that the research was conducted in the absence of any commercial or financial relationships that could be construed as a potential conflict of interest.

## Publisher’s note

All claims expressed in this article are solely those of the authors and do not necessarily represent those of their affiliated organizations, or those of the publisher, the editors and the reviewers. Any product that may be evaluated in this article, or claim that may be made by its manufacturer, is not guaranteed or endorsed by the publisher.
